# Role of machine learning algorithms in suicide risk prediction: a systematic review-meta analysis of clinical studies

**DOI:** 10.1186/s12911-024-02524-0

**Published:** 2024-05-27

**Authors:** Houriyeh Ehtemam, Shabnam Sadeghi Esfahlani, Alireza Sanaei, Mohammad Mehdi Ghaemi, Sadrieh Hajesmaeel-Gohari, Rohaneh Rahimisadegh, Kambiz Bahaadinbeigy, Fahimeh Ghasemian, Hassan Shirvani

**Affiliations:** 1 School of Engineering and the Built Environment, Anglia Ruskin University, Chelmsford, UK; 2 Health Services Management Research Center, Institute for Futures Studies in Health, Kerman University of Medical Sciences, Kerman, Iran; 3 Medical Informatics Research Center, Institute for Futures Studies in Health, Kerman University of Medical Sciences, Kerman, Iran; 4 Department of Computer Engineering, Faculty of Engineering, Shahid Bahonar University of Kerman, Kerman, Iran

**Keywords:** Machine learning, Risk prediction, Suicide prevention, Meta-analysis, Meta-synthesis

## Abstract

**Objective:**

Suicide is a complex and multifactorial public health problem. Understanding and addressing the various factors associated with suicide is crucial for prevention and intervention efforts. Machine learning (ML) could enhance the prediction of suicide attempts.

**Method:**

A systematic review was performed using PubMed, Scopus, Web of Science and SID databases. We aim to evaluate the performance of ML algorithms and summarize their effects, gather relevant and reliable information to synthesize existing evidence, identify knowledge gaps, and provide a comprehensive list of the suicide risk factors using mixed method approach.

**Results:**

Forty-one studies published between 2011 and 2022, which matched inclusion criteria, were chosen as suitable. We included studies aimed at predicting the suicide risk by machine learning algorithms except natural language processing (NLP) and image processing.

The neural network (NN) algorithm exhibited the lowest accuracy at 0.70, whereas the random forest demonstrated the highest accuracy, reaching 0.94. The study assessed the COX and random forest models and observed a minimum area under the curve (AUC) value of 0.54. In contrast, the XGBoost classifier yielded the highest AUC value, reaching 0.97. These specific AUC values emphasize the algorithm-specific performance in capturing the trade-off between sensitivity and specificity for suicide risk prediction.

Furthermore, our investigation identified several common suicide risk factors, including age, gender, substance abuse, depression, anxiety, alcohol consumption, marital status, income, education, and occupation. This comprehensive analysis contributes valuable insights into the multifaceted nature of suicide risk, providing a foundation for targeted preventive strategies and intervention efforts.

**Conclusions:**

The effectiveness of ML algorithms and their application in predicting suicide risk has been controversial. There is a need for more studies on these algorithms in clinical settings, and the related ethical concerns require further clarification.

## Highlights

• Understanding various factors associated with suicide is crucial for prevention and intervention efforts.

• Machine learning (ML) could enhance the prediction of suicide attempts.

• The neural network (NN) algorithm exhibited the lowest accuracy of 0.70.

• The random forest demonstrated the highest accuracy for suicide prediction.

## Introduction

Suicide is a global concern recognized by the World Health Organization (WHO), with a life lost to suicide every 40 s, making suicide prevention a pressing priority worldwide [1]. This form of violent death not only brings personal tragedy but also poses a significant threat to communities’ socio-psychological well-being and stability [2]. While suicide is a complex phenomenon influenced by multiple factors, behavioral, lifestyle, and clinical, can significantly contribute to an elevated risk of suicide [3]. For example, substance use can be considered a significant factor for suicide within the behavioral category [4]. Job and financial problems serve as important examples of lifestyle-related suicide risk [5]. Additionally, mental disorders are crucial clinical factors associated with suicide risk [6]. Early identification of risk factors is crucial in predicting suicide [7, 8]. The prevalence of suicide is exceptionally high among adolescents and young adults, specifically those aged 15 to 44, it is not a universal phenomenon [9]. Research indicates that, in some countries the lower risk of suicide among older individuals may be due to their enhanced resilience and greater capacity to cope with adversity, potentially reducing the likelihood of suicidal behavior [10, 11]. The other common factor can be gender. Some studies have revealed that gender differences in suicide rates indicate that men are more likely to die by suicide. However, this remains controversial because each gender is influenced by many other biological and environmental factors [12]. Suicide imposes financial burden on the healthcare system. For example, in Canada, New Zealand, and Ireland, the estimated direct and indirect costs of each suicide are approximately 443,000, 1.1 million, and 1.4 million pounds, respectively [13–15]. A comprehensive review of the works by these authors leads us to the conclusion that suicide is a global issue. Consequently, it is imperative for countries worldwide to collaborate in addressing this concern [1]. There is a growing interest in utilizing machine learning (ML) techniques for predicting suicide risk to address the issue. ML is a combination of statistical and computational models that can learn from data and improve through experience [16]. It is categorized into two main types: supervised and unsupervised. In supervised learning, the model is trained on labelled databases; however, in unsupervised learning, the model relies on unlabeled databases [17]. Both supervised and unsupervised algorithms can be utilized for suicide prediction depending on the type of database and the nature of the prediction.

Research by Walsh, Ribeiro, and Franklin (2017) demonstrated the superior performance of ML over conventional methods in accurately identifying suicide attempts [9]. ML methods have gained prominence due to their ability to extract valuable insights from diverse datasets and organize data efficiently [10, 11]. While ML shows promise in predicting suicide events, it is vital to consider the varied outcomes produced by different ML algorithms. The study conducted by various researchers suggests that while there have been notable scientific advancements in leveraging digital technologies, such as ML algorithms to prevent suicide attempts and identify at-risk individuals, there are still limitations in terms of training, knowledge, and the integration of databases [18–20]. Current suicide risk assessment methods heavily rely on subjective questioning, limiting their accuracy and predictive value [21]. As such, this study aims to systematically review previous research that has applied ML methods to predict suicide attempts and identify patients at high risk of suicide. The primary objectives are to evaluate the performance of various ML algorithms and summarize their effects on suicide. Additionally, the study aims to identify significant variables that serve as more effective suicide risk factors.

## Materials and methods

### Search strategy and study selection

We adhered to the PRISMA (Preferred Reporting Items for Systematic Reviews and Meta-Analyses) guidelines to systematically identify, select, and assess relevant studies for inclusion in our review. Our search strategy focused on PubMed, Scopus, Web of Science and SID databases, and there were no limitations on the publication date, ensuring comprehensive coverage of the literature. The project was initiated on June 1, 2022, and concluded on August 8, 2023, with a focus on two domains: machine learning (ML) and suicide.

To capture relevant studies, our search strategy incorporated keywords such as “self-harm”, “self-injury”, “self-destruction”, “self-poisoning”, “self-mutilation”, “self-cutting”, “self-burning”, “suicid*”. Additionally, we explored using artificial intelligence and ML techniques to predict suicidal attempts by employing “AND” and “OR” operators. The management of literature was facilitated through Endnote X7.

The study encompassed two primary outcomes: first, identifying the most effective ML algorithms based on their outcome measures, and second, identifying influential risk factors for predicting suicide. These outcomes were instrumental in achieving a comprehensive understanding of the field and informing our research objectives.

### Inclusion & exclusion criteria

Inclusion criteria were applied to identify relevant studies for our review. The following criteria were considered:

1. Population: Studies that included participants from various age groups, including pediatrics, geriatrics, and all-age populations, were included.
2. Language: Only studies published in the English language were included.
3. Methods: Studies employing ML methods to predict suicide were included.
4. Publication format: Studies published as journal articles, theses, and dissertations were included.
5. Study design: Various study designs, including prospective, retrospective, retrospective cohort, case-cohort, case-control, cohort, diagnostic/prognostic, longitudinal, longitudinal cohort, longitudinal prospective, prognostic, prospective cohort, retrospective, retrospective cohort, and randomized control trial studies, were considered for inclusion.

Exclusion criteria were applied to select relevant studies for our analysis. Studies were excluded if they met the following criteria:

1. Population: Studies focusing specifically on military personnel and veterans were excluded. Including military personnel and veterans in our analysis could introduce unique variables and considerations related to their distinct healthcare needs, access to services, and experiences. For example, military personnel and veterans often have specific healthcare requirements stemming from their service-related experiences. These may encompass a range of issues, including physical injuries sustained during deployment, exposure to hazardous environments leading to unique health challenges, and complex medical histories shaped by their military service.
  Moreover, their access to healthcare services can differ significantly from that of the general population. To maintain the homogeneity of our study population and to ensure the relevance and applicability of our findings to the specific context of hospitals, we have opted to exclude this subgroup.

2. Social media-based studies: Studies aiming to predict suicide attempts using ML among adults who posted or searched content related to suicide on internet platforms such as Twitter, Instagram, and Google were excluded.
3. Natural language processing (NLP) and image processing methods: Studies utilizing NLP and image processing techniques for predicting suicide attempts were excluded.
4. Publication type: Conference papers, reviews, letters to editors, book chapters, and commentary papers were excluded from the analysis.

By applying these inclusion and exclusion criteria, we aimed to select studies that align with the objectives and focus of our research.

### Data collection process

Data extraction was conducted using Microsoft Excel 2016 spreadsheet. The following information was extracted from each included study:

1. Study title: The title of the research article.
2. Authors: The names of the authors involved in the study.
3. Year of publication: The year in which the study was published.
4. Country of study: The geographical location where the study was conducted.
5. Population: The target population or participants involved in the study.
6. Type of study: The research design employed in the study.
7. Sample size: The number of participants included in the study.
8. Study objective: The main objective or aim of the study.
9. Suicide risk factors: Factors or variables considered in predicting suicide risk.
10. ML models: The specific ML models used in the study.
11. Outcome measures: Various performance metrics used to evaluate the models, including area under the curve (AUC), sensitivity, specificity, accuracy, false negative rate, false positive rate, true positive rate, negative predictive value, positive predictive value, precision, and recall.

### Quality assessment

The quality of the included articles was assessed using the Mixed Methods Appraisal Tool (MMAT 2018) following the search process. We adopted MMAT’s five sets of criteria to evaluate the quality of each type of study included in our analysis, namely qualitative, randomized controlled, nonrandomized, quantitative descriptive, and mixed methods studies [8]. This rigorous assessment process allowed us to evaluate the included studies’ methodological quality and ensure our findings’ reliability and validity.

### Data analysis methods

During the quantitative phase, the extracted data were analyzed using STATA 14.1 statistical software to conduct meta-analytic procedures. We applied the Freeman-Tukey double arcsine transformation to estimate the pooled prevalence of study outcomes and their corresponding 95% confidence intervals (CI). This transformation was also utilized to stabilize variances when generating the CIs. A random-effects model based on DerSimonian and Laird’s method was employed in the pooled data collection to account for between-study variability. This model incorporated the variability using the inverse-variance fixed-effect model [22, 23].

In the qualitative phase, the extracted data was imported into MAXQDA 20 software to facilitate meta-synthesis procedures. This critical stage involved coding the suicide risk factors from our final studies based on various themes or categories, such as demographic (demographic factors, such as age, gender, marital status), clinical and behavioral (certain behaviors, like impulsivity, self-harm, or aggression, and clinical factors involve mental health diagnoses and conditions), lifestyle (encompass aspects of an individual’s daily life, including habits and routines), laboratory and biomarkers (these could include genetic markers, hormonal imbalances), and questionnaires (the use of standardized scales and questionnaires helps quantify and measure psychological factors associated with suicide risk). Through this process, we aggregated the coded data to identify common suicide risk factors across all the studies, allowing for a comprehensive understanding of the topic.

### Publication bias

We researched various languages and databases to address study, citation and database biases. We enhanced our search strategy, resulting in the identification of 7529 publications. This abundance of sources highlights the prevalence of multiple-citation publications within our dataset. Given the common occurrence of publishing study results in similar or consecutive articles, we utilized EndNote software to identify duplicates and mitigate the risk of multiple publications and bias.

## Results

**Fig. 1:**
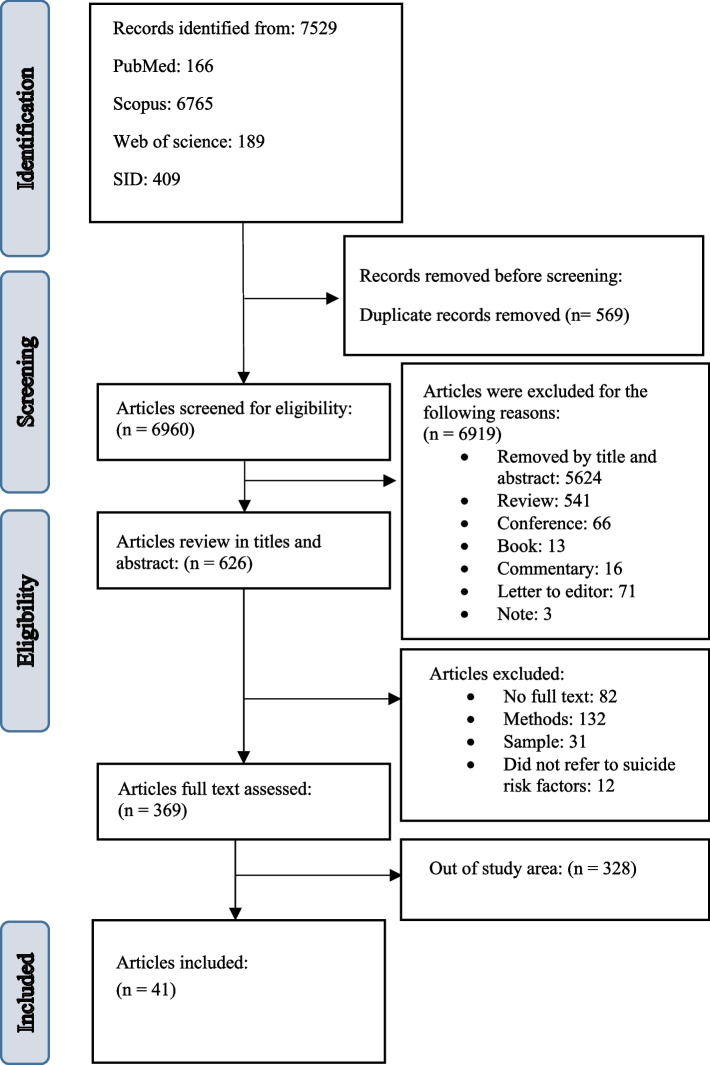
PRISMA flow diagram for the selection of studies on ML algorithms used for the purpose of suicide prediction

Figure 1 presents the PRISMA flow chart, which provides a concise review process overview. The initial search yielded 7,529 published studies. After removing 569 duplicate records, we screened the titles and abstracts of the remaining 6,624 papers. Based on this screening, 5,624 papers were excluded as they did not meet the inclusion criteria. Subsequently, the full texts of the remaining 369 studies were thoroughly assessed to determine their eligibility for inclusion in the analysis. Among these, 328 studies were deemed ineligible as they did not meet the predetermined criteria. Ultimately, 41 studies were selected for the meta-analysis and meta-synthesis, meeting the quality assessment criteria. Overall, the selected studies demonstrated satisfactory quality.

The included studies had sample sizes ranging from 159 to 13,980,570, as reported in previous research [24, 25]. The mean sample size of (Mean = 549,944.51) refers to the average number of participants across the studies included in our analysis. This value is important as it indicates the generalizability of the findings. Larger sample sizes contribute to more robust and reliable results, allowing for broader applicability of our conclusions.

Standard deviation of (SD = 2,242,858.719), reflects the variability in sample sizes observed across the individual studies. Some studies may have significantly larger or smaller sample sizes compared to the mean, resulting in a wide dispersion of values. This variability influences the heterogeneity of the overall findings and underscores the need to consider the diversity in sample sizes when interpreting the results. The median of sample size, representing the central value, is 13,420. Most of these studies were conducted in the United States and South Korea, with cohort and case-control designs being the most employed study designs. The participants in these studies predominantly represented the general population. The outcome measurement criteria of the data collection process and its results are presented below.

### Pooled prevalence of ML outcomes

Additional details of the studies included can be found in Table 1 (after reference section). Note that the statistical analysis revealed that the negative predictive value and the false positive rate did not show a significant difference, with a p-value greater than 0.05. To identify single study influence on the overall meta-analysis, a sensitivity analysis was performed using a random-effects model and the result showed there was no evidence for the effect of single study influence on the overall meta-analysis.

**Table 1:** Details of included studied, systematic review mixed-method in order to identify most common suicide risk factors and ML outcomes

Authors, published year	Country	Study design	Sample size	Participants	Most important suicide risk factors	The best ML algorithms	Outcome
(Alexopoulos et al., 2021) [59]	USA	randomized clinical trial	249	Older adults (aged 60 or above) diagnosed with major depression	Hopelessness, Neuroticism, And Low General Self-Efficacy, Avoidance/Rumination, Activation, Disability [12-Item World Health Organization Disability Assessment Schedule II (WHODAS-II), NEO Neuroticism Subscale, [Beck Hopelessness Scale (BHS), [Subscale Of The Behavioral Activation For Depression Scale (BADS)	least absolute shrinkage and selection operator (LASSO)	AUC = 0.735
(Bai et al., 2021) [24]	China	cohort	159	Individuals diagnosed with major depressive disorder who experience suicidal ideation, compared to healthy participants	Total Cholesterol (TC), Triglyceride (TG), HDLC, Low Density Lipoprotein Cholesterol (LDLC), APOA1, Apo Lipoprotein B (APOB), Lipoprotein A (LPA), Hypersensitive C-Reactive Protein (CRP), TRSF, Homocysteine (HCY), And AAT, BMI, Age, Sex, Education, Marital Status, Family History Of Mental Illness, Smoke, Hamilton Rating Scale For Depression (HDRS Scores), Beck Scale For Suicide Ideation-Chinese Version (BSI-CV Scores)	Random Forest	AUC = 0.928
(Balbuena et al.,2022) [60]	Norway, Saskatoon	retrospective cohort	185,889	General population	Income, Age, Sex, Marital Status, Month Of Birth, Living With A Spouse, Education, Social Deprivation, Mood Symptoms, Nervous Or Worried, Anxiety, Irritable, Felt Confident And Calm, Felt Happy And Optimistic, Felt Down, Depression, Felt Lonely, Smoke, Alcohol, Physical Activity, Exposure To Smoke-Filled Rooms, And Exposure To Secondary Smoke As A Child, Substance Abuse, Triglyceride, HDL-Cholesterol, Glucose, Total Cholesterol, Waist-Hip Ratio, Blood Pressure Medications, BMI, Having An Injury Requiring Hospitalization, Eating Disorder, Personality Disorder, Schizophrenia, Mania, ADHD, Repeated Episodes Of Self-Harm (RESH)	COX (male) Random Forest (female)	AUC (male): 0.57, AUC (female): 0.50
(Ballester et al.2021) [61]	Brazil	Prospective cohort study	1560	General population	Sex, Socioeconomic Status, Age, Education, Quality Of Life, Mental Disorder Symptoms	gradient tree boosting	True positive rate = 0.79, False positive rate = 0.28
(Barak-Corren et al., 2017) [62]	USA	retrospective cohort	1,724,785	Inpatient and outpatient	Mental Disorders, Personality Disorders, Schizophrenia, Psychotic Disorders, Open Wounds, Superficial Injury, Sex, Age, Race, Ethnicity, Veteran Status, Marital Status, Substance Abuse, Alcohol	Naive Bayesian classifier	AUC = 0.77, Sensitivity = 0.54, Specificity = 90%
(Bhak et al., 2019) [63]	South Korea	cohort	182	The study included individuals who had attempted suicide, patients diagnosed with major depressive disorder, and a group of healthy controls	Age, Sex, Family History Of Depression, Family History Of Suicide Attempt, Depression, Antidepressant, Hamilton Rating Scale For Depression–17, SSI Scale For Suicidal Ideation	random forest classifier	Accuracy = 92.6% in distinguishing suicide attempters from major depressive disorder patients 87.3% in distinguishing major depressive disorder patients from controls 86.7% in distinguishing suicide attempters from controls Average accuracy = 88.87
(Cho et al., 2020) [64]	South Korea	cohort	372,813	Individuals who are subscribers of health insurance or recipients of medical aid and have been referred for a check-up	Y-Glutamyl Trans peptidase, BMI, Demographics: Age, Exercise, Alcohol	random forest	AUC during follow-up = 0.849, AUC after follow-up = 0.818, Average AUC = 0.8335, positive predictive value during follow-up = 0.007, positive predictive value after follow-up = 0.003, Average positive predictive value = 0.005, negative predictive value during and after follow-up = 0.999, Average negative predictive value = 0.999, False negative rate during follow-up = 0.183, False negative rate after follow-up = 0.341, Average False negative rate = 0.262, False positive rate during follow-up = 0.247, False positive rate after follow-up = 0.212, Average False positive rate = 0.2295 , accuracy during follow-up = 0.754, accuracy after follow-up = 0.788, Average accuracy = 0.771, Sensitivity during follow-up = 0.817, Sensitivity after follow-up = 0.657, Average sensitivity = 0.737, Specificity during follow-up = 0.754, Specificity after follow-up = 0.788 Average Specificity = 0.771
(Cho et al., 2021) [42]	South Korea	cohort	48,047	Individuals who underwent health screening	Sex, Insurance, Age, Depression, Antidepressants, Aspartate Transaminase, Waist Circumference, Sleeping Pills, BMI, Benzodiazepine, Glucose, Cholesterol, Lipoprotein (HDL), Hemoglobin, Triglyceride, Systolic Blood Pressure, Low-Density Lipoprotein (LDL), Creatinine, Y-Glutamyl Transpeptidase	random forest	AUC = 0.818, positive predictive value = 0.007, negative predictive value = 0.999, Accuracy = 0.832, Sensitivity = 0.600, Specificity = 0.833
(Choi et al., 2021) [50]	South Korea	longitudinal study	78,796	General population	Income, Age, Drinking Age, Average Sleep Time Per Day, Education, Home Ownership, Economic Activity, Uncomfortable Experience In Past 2 Weeks, Sex, Depression, Anxiety, Athletic Ability, Pain/Discomfort	XGBoost classifier	AUC suicidal ideation = 0.950 AUC suicide attempts = 0.990 Average AUC = 0/97, Precision suicidal ideation = 0.874, Precision suicide attempts = 0.977, Average precision = 0.9255 , Recall suicidal ideation = 0.893, Recall suicide attempts = 0.986 Average recall = 0/9395, Accuracy suicidal ideation = 0.893, Accuracy suicide attempts = 0.986, Average accuracy = 0.9395
(Kyu Sung Choi et al., 2021) [65]	South Korea	longitudinal study	31,720	General population	Age, Sex, Anxiety, Resilience, Self-Esteem, Depression, Neuropsychiatric Disorder, Patient Health Questionnaire-9 (PHQ-9), Generalized Anxiety Disorder-7 (GAD-7), State-Trait Anxiety Inventory-State Anxiety (STAI-S, Or STAI-X1), Resilience Appraisal Scale (RAS), Rosenberg Self-Esteem Scale (RSES), Mini International Neuropsychiatric Interview (MINI)	deep graph neural network model (GIN)	AUC = 0.878, Sensitivity = 76.3, Specificity = 83.4
(Choi et al., 2018) [46]	South Korea	cohort	819,951	Korean who are covered by Medicare and Medicaid insurance inpatient or outpatient	Disability, Endocrine, Nutritional And Metabolic Diseases, Mental And Behavioral Disorders, Diseases Of The Nervous System, Diseases Of The Eye And Adnexa, Diseases Of The Ear And Mastoid Process, Diseases Of The Respiratory System, Diseases Of The Genitourinary System, Injury, Poisoning, Factors Influencing Health Status, Sex, Age, Type Of Insurance, Household Income	Cox regression	AUC = 0.688
(R. Yates Coley et al., 2021) [25]	USA	diagnostic/prognostic study	13,980,570	Outpatient appointments with a mental health professional	Bipolar, Schizophrenia, Mental Disorder Symptoms, Encounters In Past 5 Y With Mental Health Diagnosis, Depression, Anxiety, Race, Sex, Age, Medicaid Insurance, Alcohol, Substance Abuse, Patient Health Questionnaire 9 (PHQ-9 Score)	Logistic regression with LASSO	AUC = 0.822
(Edgcomb et al., 2021) [43]	USA	longitudinal cohort study	15,644	Adults with serious mental illnesses are at high risk for both medical illness and serious suicide attempts and self-harm	Medical Comorbidity, Age, Number of Medical Hospitalizations In The Previous Year, Alcohol, Bipolar, In-Hospital Mortality Score, Walraven Score	Classification and Regression Tree algorithm (CART)	AUC = 0.86
(Edgcomb et al., 2021) [31]	USA	retrospective cohort study	142,476	women with depression, bipolar disorder, schizophrenia or schizoaffective disorder	Medical Comorbidity, History of Pregnancy-Related Mental Illness, Age, Van Walraven Score	Classification and Regression Tree algorithm (CART)	AUC repository1 = 0.73, AUC repository2 = 0.71, Average AUC = 0.72 , Accuracy repository1 and repository2 = 0.84, Sensitivity repository1 = 73.4, Sensitivity repository2 = 83.3, Average Sensitivity = 78.35 , Specificity repository1 = 84.1, Specificity repository2 = 82.2, Average Specificity = 83.15
(Etter et al., 2017) [66]	USA	Prospective cohort study	2134	General population	Sex, Race, Age, Substance Abuse	Logistic regression	AUC = 0.84
(Ge et al., 2020) [67]	China	retrospective study	1916	patients with major depressive disorder	Age, Sex, Marital Status, Vocational Status, Ethnicity, Depression, Anxiety, Free Triiodothyronine (FT3), Free Thyroxine (FT4), Thyroxine, Triiodothyronine, Thyrotropin Stimulating Hormone (TSH), Hamilton Depression Scale (HAMD), Hamilton Anxiety Rating Scale (HAMA)	neural network	AUC = 0.76, Accuracy = 70.08, Sensitivity = 70.68, Specificity = 67.09
(Hill et al., 2019) [68]	USA	longitudinal study	4834	General population	HIV/STD/Sex-Related, Depression, Risky Behavior, Relationship, Friends Have Succeeded in Committing Suicide In The Past 12 Months, Resident Mother, Race, Substance Abuse	A Classification Tree Approach (CTA)	Accuracy Wave 1 = 85.1, Accuracy Wave 2 = 71.7, Average accuracy = 78.4, Sensitivity Wave 1 = 69.8, Sensitivity Wave 2 = 90.6, Average sensitivity = 80.2, Specificity Wave 1 = 85.7, Specificity Wave 2 = 70.9, Average specificity = 78.3
(Kim et al., 2021) [69]	South Korea	retrospective analysis	7824	Students who have been referred for a health check-up	Family Problems, Interpersonal Passivity, Aberrant Experiences, Social Avoidance, Shyness, Disaffiliativeness, Juvenile Conduct Problems, Aggression, Activation, Hopelessness, Helplessness, Self-Doubt, Inefficacy, Stress/Worry, Anxiety, Anger Proneness, Behavior-Restricting Fears, Multiple Specific Fears, Malaise, Demoralization, Low Positive Emotions, Cynicism, Antisocial Behavior, Ideas Of Persecution, Dysfunctional Negative Emotions, Hypomanic Activation, Emotional/Internalizing Dysfunction, Thought Dysfunction, Behavioral/Externalizing Dysfunction, Substance Abuse, Gastro-Intestinal Complaints, Head Pain Complaints, Neurological Complaints, Cognitive Complaints, Somatic Complaints, Suicidal/Death Ideation (SUI) Scale, PSY-5 (Personality Psychopathology Five) Scales, AES Aesthetic-Literary Interests, MEC Mechanical-Physical Interest	Random forest	AUC suicide ideation = 0.844, AUC suicide attempt = 0.851, Average AUC = 0.8475 , Precision suicide ideation = 0.920, Precision suicide attempt = 0.953, Average precision = 0.9365 , Recall suicide ideation = 0.929, Recall suicide attempt = 0.950, Average recall = 0.9395 , Accuracy suicide ideation = 92.9, Accuracy suicide attempt = 95 , Average accuracy = 93.95 ,
(Haroz et al., 2022) [70]	USA	retrospective cohort	13,420	Pediatric patients	Sex, Age, Race, Anxiety, Depression, Alcohol, Obesity, Tobacco, ADHD, Brain Injury, Psychotic Disorders, Anemia, Bipolar, Migraine, Autism, Diabetes, Drug, Asthma, Learning Disorder, Intellectual Disability, PTSD, Epilepsy, Cancer, Hemiplegia, Cerebrovascular Disease, Other Developmental Disorder, Renal Disease, Mild Liver Disease, Rheumatoid Disease, Heart Failure, Vascular Disease, Metastatic Tumor, AIDS, Severe Liver Disease, Myocardial Infraction, Leukemia And Lymphoma, Personality Disorder, Charlson Comorbidity Index	Binary Logistic Regression model	AUC Model 2: ASQ + HER = 0.843, positive predictive value Model 2: ASQ + HER = 0.103, False negative rate Model 2: ASQ + HER = 0.183, Accuracy Model 2: ASQ + HER = 0.815, Sensitivity Model 2: ASQ + HER = 0.817
(Melhem et al., 2019) [71]	USA	longitudinal study	663	offspring of parents (or probands) with mood disorders	Bipolar, Posttraumatic Stress, Hyperactivity, Childhood Abuse, Psychotherapy, Antidepressants, Nonpsychotropic Medications, Hopelessness, Impulsivity, Impulsive Aggression, Irritability, Mood Disorders, Depression, Anxiety, Aggression, Sex, Age, Race, Income, Alcohol, Substance Abuse	logistic regression	AUC = 0.80, Sensitivity = 87.3, Specificity = 63
(Machado et al., 2020) [72]	USA	Prospective cohort study	77,746	General population	BMI, Psychotic Disorders, Personality Disorders, Mood Disorder, Anxiety, Age, Sex, Race, Marital Status, Education, Income, Substance Abuse	Elastic net regularization	AUC distinguished individuals who had attempted suicide from those who had not = 0.89, AUC participants with lifetime major depressive episodes = 0.89, Average AUC = 0.89, positive predictive value distinguished individuals who had attempted suicide from those who had not = 0.0455, positive predictive value participants with lifetime major depressive episodes = 0.1048, Average positive predictive value = 0.07515 , negative predictive value distinguished individuals who had attempted suicide from those who had not = 0.9980, negative predictive value participants with lifetime major depressive episodes = 0.9944, Average negative predictive value = 0.9962 , Sensitivity distinguished individuals who had attempted suicide from those who had not = 0.7451, Sensitivity participants with lifetime major depressive episodes = 0.7742, Average sensitivity = 0.75965 , Specificity distinguished individuals who had attempted suicide from those who had not = 0.8922, Specificity participants with lifetime major depressive episodes = 0.8586, Average specificity = 0.8754
(Miranda et al., 2022) [73]	USA	retrospective study	38,807	posttraumatic stress disorder (PTSD) patients, a population with a high risk of suicide	Glucose, Chloride, Red Cell Distribution Width, Mean Corpuscular Hemoglobin, Hemoglobin, Hematocrit, Mean Corpuscular Volume, White Blood Cell, Anion Gap, Blood-Urine, ABS Neutrophils, Glucose-Urine, Glucose (Bedside Test), ABS Basophils, Red Blood Cell, Potassium, International Normalized Ratio, Ionized Calcium ISTAT, Ionized Calcium, Bacteria, Base Excess, Total Protein, Calcium, Red Blood Cells-Urine, Sodium, Prothrombin Time, Mean Platelet Volume, Platelets	deep-learning (DRNN)	AUC = 0.932
(Zheng et al., 2020) [74]	USA	cohort	236,347	Patients who visited three specified health centers	Age, Sex, Family Problems, Unemployment, Anxiety, Depression, Mood Disorder, Anxiety, Substance Abuse, Alcohol, Tobacco, Arthritis, Back Pain, Vehicle Accidents, Head Pain Complaints, PTSD, Esophageal Disorders, Hypertension, Esophageal Reflux, Serotonin Reuptake Inhibitor, Physical Restraints, Educational Circumstances Problems, Injury, Poisoning, Psychosocial Issues, Drug Use, Asthma, Schizophrenia, Joint Pain, Nervous System Disorders, Neuropathy, Bipolar, Developmental Disorders, Fibromyalgia, Impulse Control Disorders, Personality Disorders, Endocrine Disorders, Forearm, Migraines, Borderline Personality Disorder	Deep Learning model	AUC 1-year suicide attempt = 0.792, AUC retrospective and prospective cohorts = 0.769, Average AUC = 0.7805
(Zhu et al., 2020) [75]	China	cohort	169	inpatients (Patients With Bipolar II Disorder)	Age, Education, Sex, Marital Status, Family History Of Mental Disorder, Family History Of Suicide, Occupation, Seasonal Characteristics, Depression, Hypomania, Substance Abuse, Course Of Disease, Combined Somatic Disorder, Psychotic Characteristics, Hamilton Depression Rating Scale (Scores Of HAMD-17), Hamilton Depression Rating Scale (Scores Of HAMD-16), Hamilton Anxiety Rating Scale (Scores Of HAMA), Nurses’ Global Assessment Of Suicide Risk (NGASR)	support vector machines (SVMs)	Accuracy = 0.84
(Setoyama et al., 2016) [76]	Japan	cohort	339	psychiatric patients	Blood Plasma: 3-Hydroxybutyrate (3HB), Betaine, Citrate, Creatinine, And Gamma-Aminobutyric Acid (GABA), Hamilton Rating Scale For Depression (HAMD)-17, Patient Health Questionnaire (PHQ)-9	logistic regression	AUC = 0.75
(Van Mens et al., 2020) [77]	Scotland	longitudinal study	3508	General population	Burdensomeness, Mental Disorder Symptoms, Impulsivity, Defeat, Entrapment, Internal Entrapment, Interpersonal Needs, Depression, External-Entrapment, Stress, Wellbeing, Twarted-Belonginess, Optimism, Perfectionism, Resilience, Interpersonal Needs, Lack of Social Support, Reengagement, Acquired Capability, Goal Disengagement, Age, Sex	random forest	AUC = 0.83, positive predictive value = 0.52
(Su et al., 2020) [78]	USA	retrospective study	41,721	pediatric population who visited specified health center	Sex, Age, Race, Ethnicity, Depression, Anxiety, Mood Disorder, Problems Related To Lifestyle, Emotional State, ADHD, Conduct Disorder, Bipolar, Personal History Of Certain Other Disease, Joint Disorder, Sertraline, Hydroxyzine Pamoate, Ethinyl Estradiol, Lorazepam, Escitalopram, Citalopram, Fluoxetine, Cholecalciferol, Hydroxine Hcl, Venlafaxine, Guanfacine, Trazodone, Prozac, Risperidone, Zoloft, Aripiprazole, Quetiapine, Culture Urine, Acetaminophen Level, Urinalysis With Microscopic, POC Pregnancy Urine, MDMA, POC Urinalysis Dipstick, Urinalysis With Microscopic, POC Pregnancy Urine, Salicylate Level, GC/Chlamydia, Oxycodone Urine	logistic regression	AUC = 0.86
(Forkmann et al., 2020) [79]	Germany	longitudinal prospective study	308	The study focused on participants over the age of 18 who were admitted to a psychiatric hospital due to severe suicidal ideation or a suicide attempt within two weeks of admission	Burdensomeness, Belongingness, Depression, Pessimistic And Hopeless Cognitions, Fearlessness About Death, Pain Tolerance, Capability For Suicide, Self-Injurious Thoughts And Behaviors Interview (SITBI), German Capability For Suicide Questionnaire (GCSQ), Interpersonal Needs Questionnaire (INQ), Rasch-Based Depression Screening (DESC-I), Beck Hopelessness Scale (BHS), Beck Scale For Suicide Ideation (BSS) (Interpersonal Theory Of Suicide, Pos. 4–8)	logistic regression	AUC = 0.729
(Adams et al., 2021) [80]	Denmark	Case–cohort study	15,953	Case: all hospital outpatient clinic visits were reported to the Danish National Patient Registry Control: all persons born or legally residing in Denmark on 1 January 1995	Sedative, Hypnotic, Anxiolytic, Age, Marital Status, Income, Immigrant, Alcohol, Opioid, Cannabis, Cocaine, Hallucinogen, Nicotine, Other Psychoactive Substance	random forests	AUC men = 0.77, AUC women = 0.86, Average AUC = 0.815
(Barak-Corren et al., 2020) [81]	USA	case control	3,714,105	Case: Cases were defined as individuals having at least 1 captured suicide attempts, Control: candidate that are likely to capture suicide attempts	Sex, Race, Ethnicity, Poisoning By Unspecified Drug Or Medicinal Substance, Drug Withdrawal Syndrome, Cocaine Dependence, Unspecified Use, Drug-Induced Organic Affective Syndrome, Borderline Personality, Unspecified Use, Unspecified Neurotic Disorder, Unspecified Use, Multiple And Unspecified Open Wound Of Upper Limb, Without Mention Of Complication, Unspecified Use, Open Wound Of Upper Arm, Without Mention Of Complication, Unspecified Use, Open Wound Of Hand Except Fingers Alone With Tendon Involvement, Assault By Cutting And Piercing Instrument, Bipolar Affective Disorder, Unspecified Degree, Unspecified Personality Disorder, Bipolar Affective Disorder, Mixed, Mixed, Or Unspecified Drug Abuse, Acute Alcoholic Intoxication In Alcoholism (Alcohol), Unspecified Drinking Behavior, Cocaine Abuse, Opioid Type Dependence, Unspecified Drug Dependence, Depression, Antipsychotics, Antidepressants, And Mood Stabilizers (Eg, Lithium), Antiretroviral Medications Used To Treat HIV/AIDS (Eg, Ritonavir) Were Associated With An Increased Risk For A Suicide Attempt. Nicotine Replacement Therapy Also Consistently Emerged As A Leading Predictor Across The Nonpediatric Hospitals, Toxicology Tests, Toxicology Screening Tests, Choriogonadotropin-Beta, Levels Of Valproic Acid, BCH, Lithium Level	naive Bayes classifiers	AUC = 0.73
(Barros et al., 2017) [82]	USA	case control	707	Case: mental health patients with suicide risk, Control: mental health patients without suicide risk	Bipolar, Mixed Episode, Adjustment Disorder, Dysthymia, Depression, Anxiety, Age, Sex, Marital Status, Parental Status, Education, Occupation	support vector machine	Accuracy = 0.78, Sensitivity = 0.77, Specificity = 0.79
(Berkelmans et al., 2021) [53]	Netherland	case control	602,270	Case: The people who died by suicide, Control: people who did not die due to suicide	Age, Sex, Income, Education, Immigration Background, Urban city, Place In Household, Healthcare Costs, Province, Social Benefits, Mental Disorder Symptoms, Physical Health Problems	logistic regression	AUC = 0.77
(Delgado-Gomez et al., 2016) [83]	Spain	case control	902	Case 1: first-time suicide attempters, Case 2: psychiatric inpatients without current or past history of suicidal behavior, Control: healthy controls	Sex, Age, Marital Status, Education, Employment Status, Suicidal Risk, Personality and Life Events Items and The Corresponding Samejima’s Parameters (PLE Scale Item)	Decision tree	Precision = 0.89, Sensitivity = 0.89, Specificity = 0.89
(D. Delgado-Gomez et al., 2011) [84]	Spain	case control	879	control: healthy blood donors, case: psychiatric inpatients	Barrat’s Impulsiveness Scale Version 11 (BIS-11), International Personality Disorder Evaluation Screening Questionnaire (IPDESQ)	Support vector machine	Accuracy = 80.26
(D. Delgado-Gomez et al., 2012) [85]	Spain	case control	883	Case: Adults with suicide attempt, Control: adults without suicide attempt	Age, Holmes–Rahe Social Readjustment Rating Scale (SRRS Questionnaire), International Personality Disorder Examination Screening Questionnaire (IPDE-SQ Questionnaire)	multivariate classification techniques	Accuracy = 0.79, Sensitivity = 78.30, Specificity = 0.91
(DelPozo-Banos et al., 2018) [86]	United Kingdom	Case–cohort study	54,684	Case: those who died by suicide, Control:	Prescription Of Psychotropic, Depression, Anxiety, Self-Harm, Age, Sex, Alcohol, Drug Abuse	ANN	Sensitivity = 64.57, Specificity = 81.86
(Gradus et al., 2021) [87]	Denmark	Case–cohort study	288,157	Case: Cases were all persons who made a nonfatal suicide attempt, Control: 5% random sample of the population at risk	Psychiatric Medications, Poisoning, Stress, Substance Abuse	Random forest	AUC (male): 0.89, AUC (female): 0.91, Average AUC: 0/9
(Jiang et al., 2021) [30]	Denmark	Case–cohort study	14,737	Cases: all persons who died by suicide and had an incident depression diagnosis, Control: 5% random sample of all individuals in Denmark	Mental Disorders, Somatic Disorders, Surgeries, Medications, Age, Marital Status, Immigrant, Family Suicide Death History, Employment, Income	Decision tree and random forest	AUC decision tree (male): 0.71, AUC random forest (female): 0.75, Average AUC: 0.73
(Harman et al., 2021) [88]	USA	case control	11,369	Case: individuals with suicidal ideation and/or attempt, Control: healthy controls	Impulsivity, Prodromal Psychosis, Loneliness, Worthlessness, Behavioral Problems	random forest	AUC: 0.77
(Navarro et al., 2021) [89]	Canada	prognostic study	1623	General population	Mother and Father Education and Mother and Father Age, Socioeconomic Status, Neighborhood Characteristics, Parents’ Psychological State, Parents’ Antisocial Behaviors, Parenting Practices, Sex, Prematurity	Random forest	AUC (male): 0.62, AUC (female): 0.72, Average AUC: 0.67 , positive predictive value (male): 0.62, positive predictive value (female): 0.60, Average positive predictive value: 0.61 , negative predictive value (male): 0.71, negative predictive value (female): 0.75, Average negative predictive value: 0.73 , Sensitivity (male): 0.32, Sensitivity (female): 0.50, Average sensitivity: 0.41, Specificity (male): 0.82, Specificity (female): 0.76, Average Specificity: 0.79
(McKernan et al., 2019) [90]	USA	case control	8879	Case: fibromyalgia patients with suicide ideation and attempt, Control: general population	Sex, Race, Age, Comorbid Medical Illness, Drug Dependence, Inpatient Utilization, Mental Disorder Symptoms, Obesity, Polysomatic Complaints	bootstrapped L-1 penalized regression	AUC suicide ideation: 0.8, AUC suicide attempt: 0.82, Average AUC: 0.81, Recall suicide ideation: 0.14, Recall suicide attempt: 0.08, Average recall: 0.11

### Accuracy

**Fig. 2:**
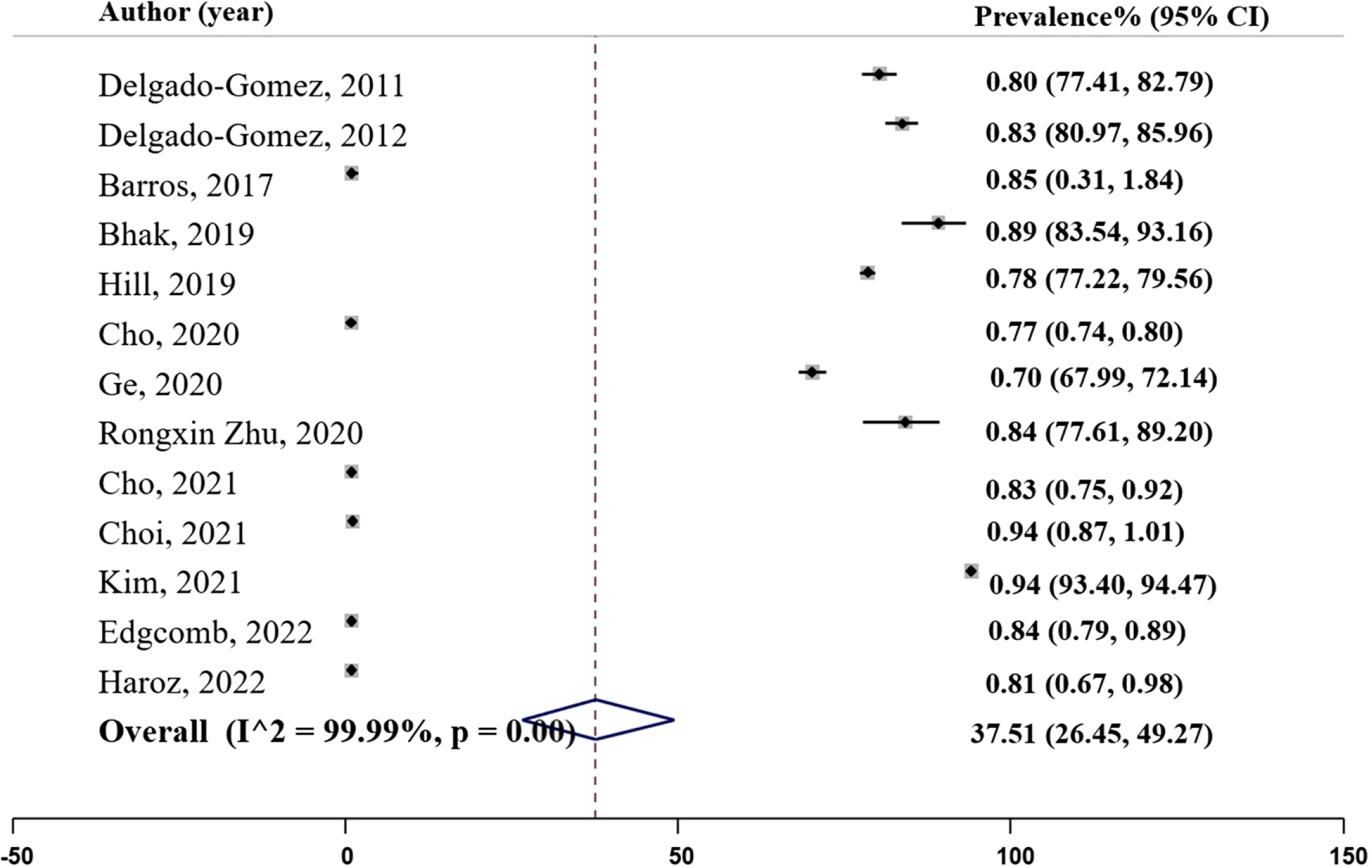
Panel A. Accuracy of the machine learning models; N studies = 13

Accuracy refers to the ability of ML models to differentiate between health and patient groups [56] correctly. Out of the 41 final studies, 13 studies reported information on accuracy. The reported accuracy rates varied across the studies as indicated in Panel A of Fig. 2, with the lowest being 0.70 for NN in the study conducted in [30], and the highest being 0.94 for the random forest in the study conducted in [32]. The overall pooled prevalence of accuracy was 0.78 ((${I}^{2}=56.32\%$; 95% CI: 0.73, 0.84), Table 2.

**Table 2:** The list of different ML outcomes, along with the pooled estimates for those outcomes that have sufficient records

ML performances	References	Pooled % (95% CI)
AUC	32 studies: *(Setoyama et al., 2016*; *Barak-Corren et al., 2017, 2020*; *Etter et al., 2017*; *Choi et al., 2018, 2021, 2021*; *Melhem et al., 2019*; *McKernan et al., 2019*; *Cho et al., 2020, 2021*; *Forkmann et al., 2020*; *Ge et al., 2020*; *Machado et al., 2020*; *Zheng et al., 2020; Van Mensa et al., 2020*; *Su et al., 2020*; *Alexopoulos et al., 2021*; *Bai et al., 2021*; *Coley et al., 2021*; *Kim et al., 2021*; *Adams et al., 2021*; *Berkelmans et al., 2021*; *Gradus et al., 2021*; *Jiang et al., 2021*; *Harman et al., 2021*; *Navarro et al., 2021*; *Balbuena et al., 2022*; *Beni Edgcomb et al., 2022, 2022*; *Haroz, E. E. et al., 2022*; *Miranda et al., 2022)*	**0.77 (0.74, 0.80)**
Accuracy	13 studies: *(Delgado-Gomez et al., 2011*, *2012*; *Barros et al., 2017*; *Bhak et al., 2019*; *Hill et al., 2019*; *Cho et al., 2020*, *2021*; *Ge et al., 2020*; *Zhu et al., 2020*; *Choi et al., 2021*; *Kim et al., 2021*; *Beni Edgcomb et al., 2022*; *Haroz, E. E. et al., 2022)*	**0.78 (0.73, 0.84)**
Sensitivity	15 studies: *(Delgado-Gomez et al., 2012, 2016*; *Barak-Corren et al., 2017, Barros et al., 2017*; *DelPozo-Banos et al., 2018*; *Hill et al., 2019*; *Melhem et al., 2019*; *Cho et al., 2020, 2021*; *Ge et al., 2020*; *Machado et al., 2020*; *Choi et al., 2021*; *Navarro et al., 2021*; *Beni Edgcomb et al., 2022*; *Haroz, E. E. et al., 2022)*	**0.69 (0.60, 0.78)**
Specificity	15 studies: *(Delgado-Gomez et al., 2012, 2016*; *Barak-Corren et al., 2017*; *Barros et al., 2017*; *DelPozo-Banos et al., 2018*; *Hill et al., 2019*; *Melhem et al., 2019; Cho et al., 2020, 2021*; *Ge et al., 2020*; *Machado et al., 2020*; *Choi et al., 2021*; *Navarro et al., 2021*; *Beni Edgcomb et al., 2022*; *Haroz, E. E. et al., 2022)*	**0.81 (0.77, 0.86)**
Positive predictive value	6 studies: *(Cho et al., 2020*; *Machado et al., 2020*; *Van Mensa et al., 2020*; *Cho et al., 2021*; *Navarro et al., 2021*; *Haroz, E. E. et al., 2022)*	**0.10 (0.04, 0.19)**
Recall	3 studies: *(McKernan et al., 2019*; *Choi et al., 2021*; *Kim et al., 2021)*	**0.58 (0.15, 1.29)**
Precision	3 studies: *(Delgado-Gomez et al., 2016*; *Choi et al., 2021*; *Kim et al., 2021)*	**0.91 (0.85, 0.98)**
False negative rate	2 studies: *(Cho et al., 2020*; *Haroz, E. E. et al., 2022)*	**0.26 (0.24, 0.28)**
True positive rate	1 study: (*Ballester et al.2021*)	**0.77 (0.40, 1.34)**

### AUC

**Fig. 3:**
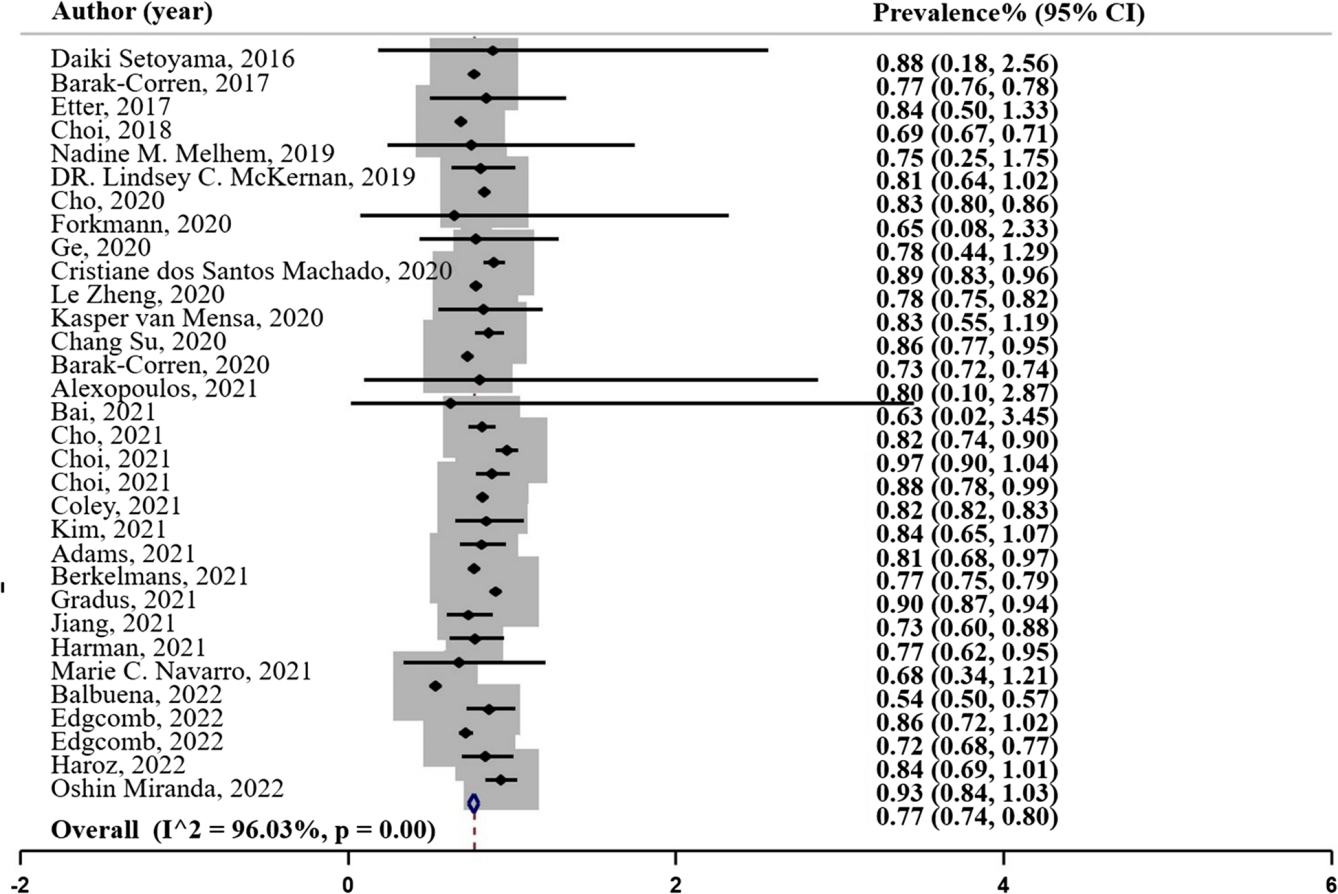
Panel B. AUC of the machine learning models; N studies = 32

The area under the curve (AUC) as a metric used in this study to compare the performance of multiple classifiers [26]. In our analysis, a total of thirty-two studies reported AUC values as indicated in Fig. 3, Panel B. Balbuena et al.’s (2022) study reported the lowest AUC of 0.54, based on the COX and random forest models. On the other hand, Choi et al.’s (2021) reported the highest AUC of 0.97, using the XGBoost classifier. The pooled prevalence of AUC across the studies was estimated to be 0.77 (${I}^{2}=$ 95.86%; 95% CI: 0.74, 0.80), Table 2.

### Precision

**Fig. 4:**
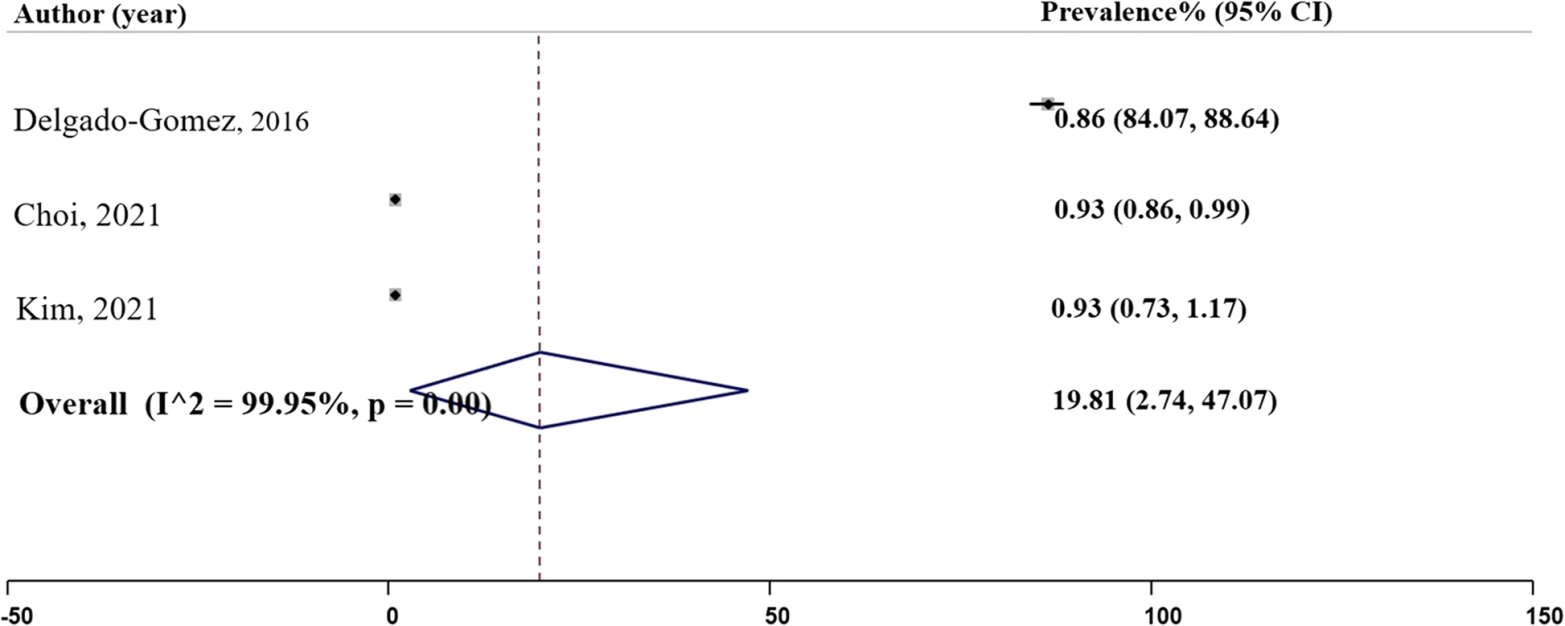
Panel C. Precision of the machine learning models; N studies = 3

Precision is a measure that determines the number of true positives divided by the sum of true positives and false positives [27]. In our analysis, three studies reported precision values as depicted in Fig. 4, Panel C. Two studies reported the highest precision rate of 0.93. The first study, conducted by Choi et al., utilized the XGBoost classifier, and the second study, by Kim et al. (2021), employed a random forest model. On the other hand, the lowest precision rate of 0.86 was documented in the Delgado-Gomez et al. (2016) study, which used a decision tree model. The pooled prevalence of positive predictive value was estimated to be 0.91 (${I}^{2}=$ 0.001%; 95% CI: 0.85, 0.98), Table 2.

### Positive predictive value

**Fig. 5:**
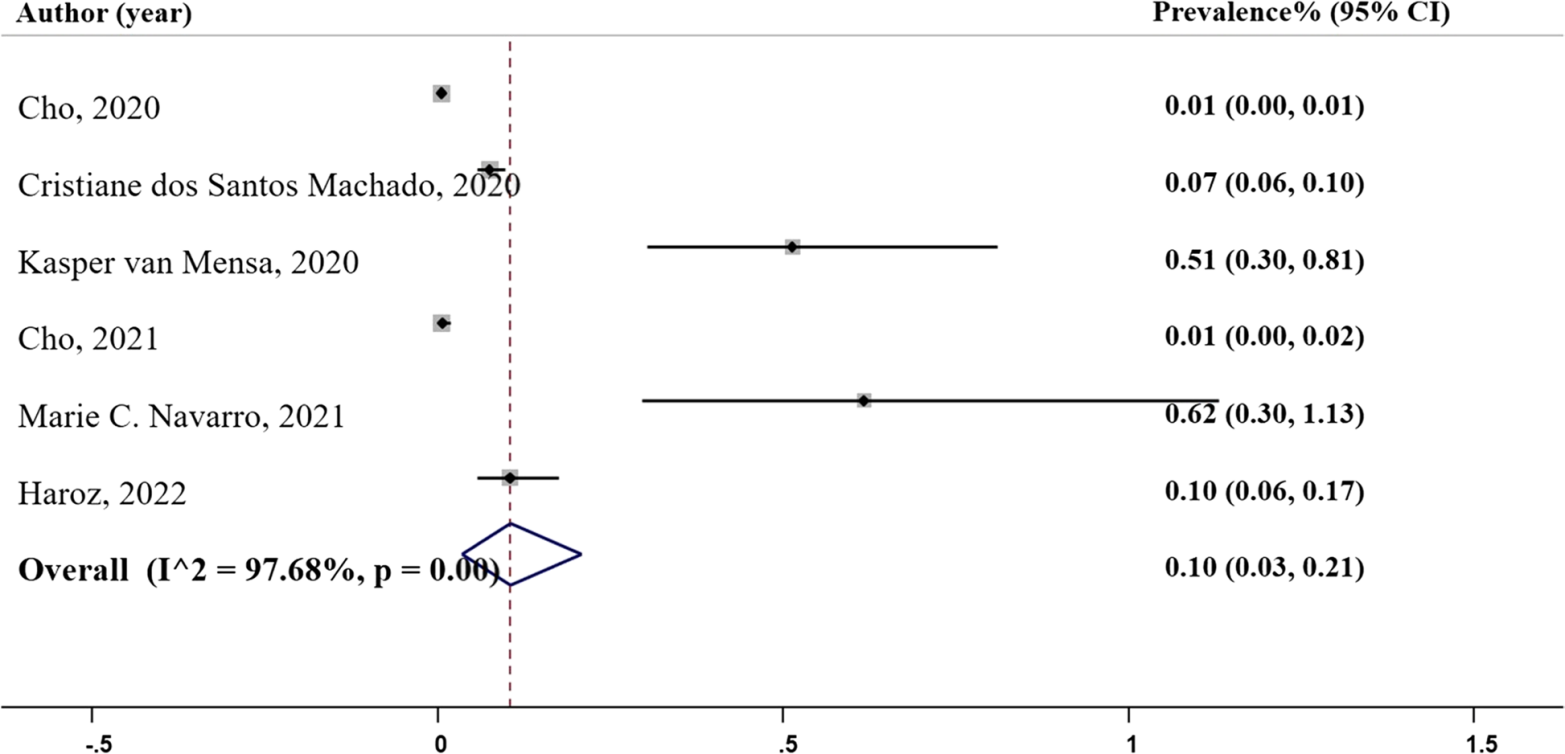
Panel D. Positive predictive value of the machine learning models; N studies = 6

Positive predictive value (PPV) represents the proportion of true positive cases among all positive predictions [27]. Among the studies included in our analysis, six studies reported PPV values as depicted in Fig. 5, Panel D. The PPV varied across the studies, ranging from 0.01 in Cho et al.’s study conducted in 2020 and 2021, which utilized a random forest model, to 0.62 in Navarro’s (2021) study, also employing a random forest model. The pooled prevalence of PPV was estimated to be 0.10 (${I}^{2}=$ 97.02%; 95% CI: 0.03, 0.21), Table 2.

### True positive rate

**Fig. 6:**
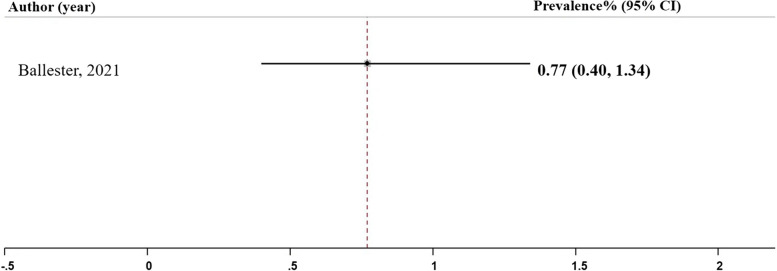
Panel E. True positive rate of the machine learning models; N studies = 1

The true positive rate (TPR), also known as sensitivity, represents the proportion of actual positive cases correctly identified by the model [27]. In our analysis, only one study conducted by Ballester et al. (2021), utilized the gradient tree boosting model, reported the TPR as depicted in Fig. 6, Panel E. The pooled prevalence of TPR in this study was estimated to be 0.77 (95% CI: 0.40, 1.34), Table 2.

### Sensitivity

**Fig. 7:**
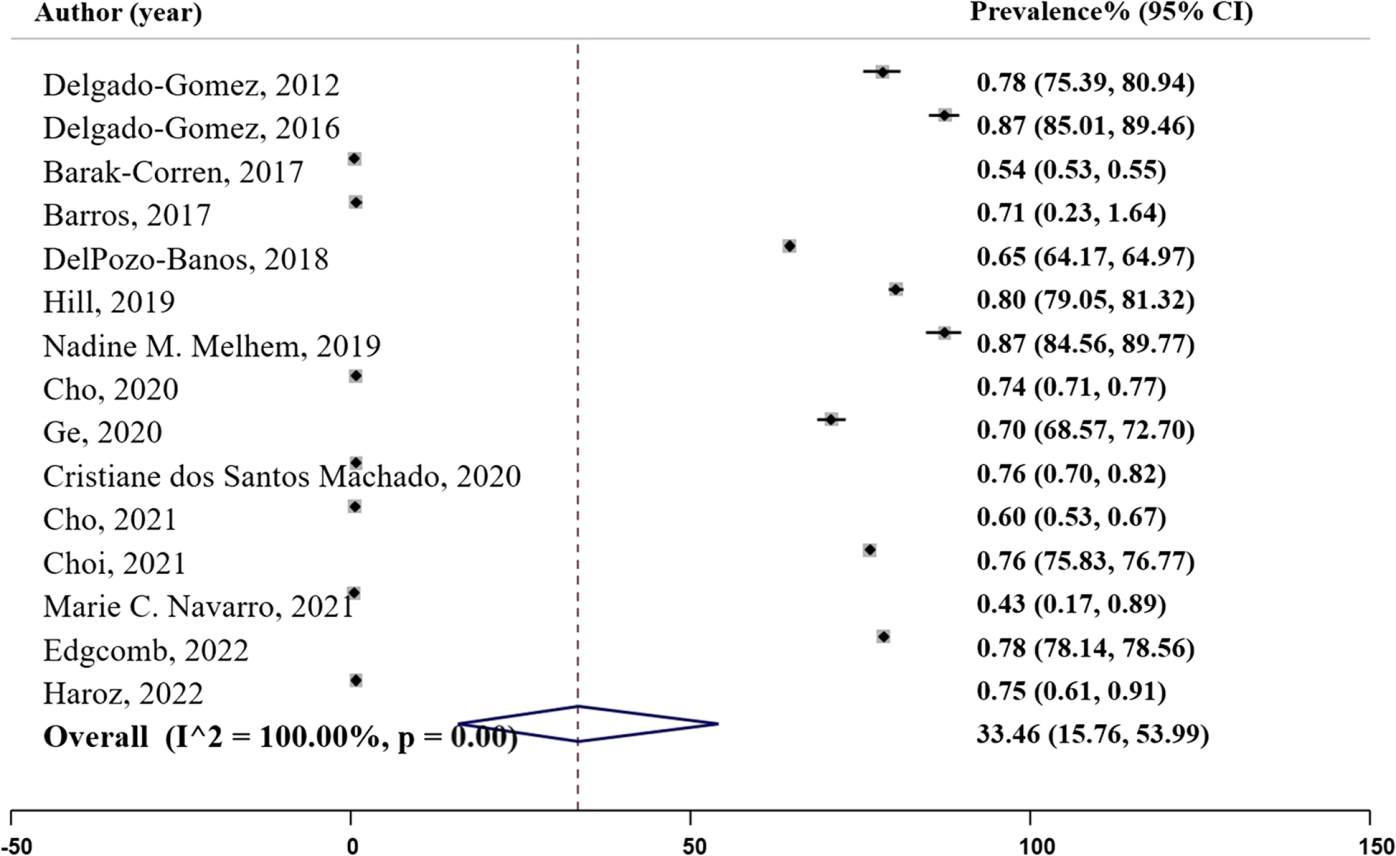
Panel F. Sensitivity of the machine learning models; N studies = 15

Sensitivity, also known as the true positive rate, measures the proportion of actual positive cases correctly identified by the model (specified patient cases) [28]. In our analysis, fifteen studies provided data on sensitivity as illustrated in Fig. 7, Panel F. The sensitivity ranged from 0.43 in Navarro’s (2021) random forest study to 0.87 in Delgado-Gomez et al.’s (2016) decision tree study. The pooled prevalence of sensitivity was estimated to be 0.69 (${I}^{2}=$ 95.94%; 95% CI: 0.60, 0.78), Table 2.

### Specificity

**Fig. 8:**
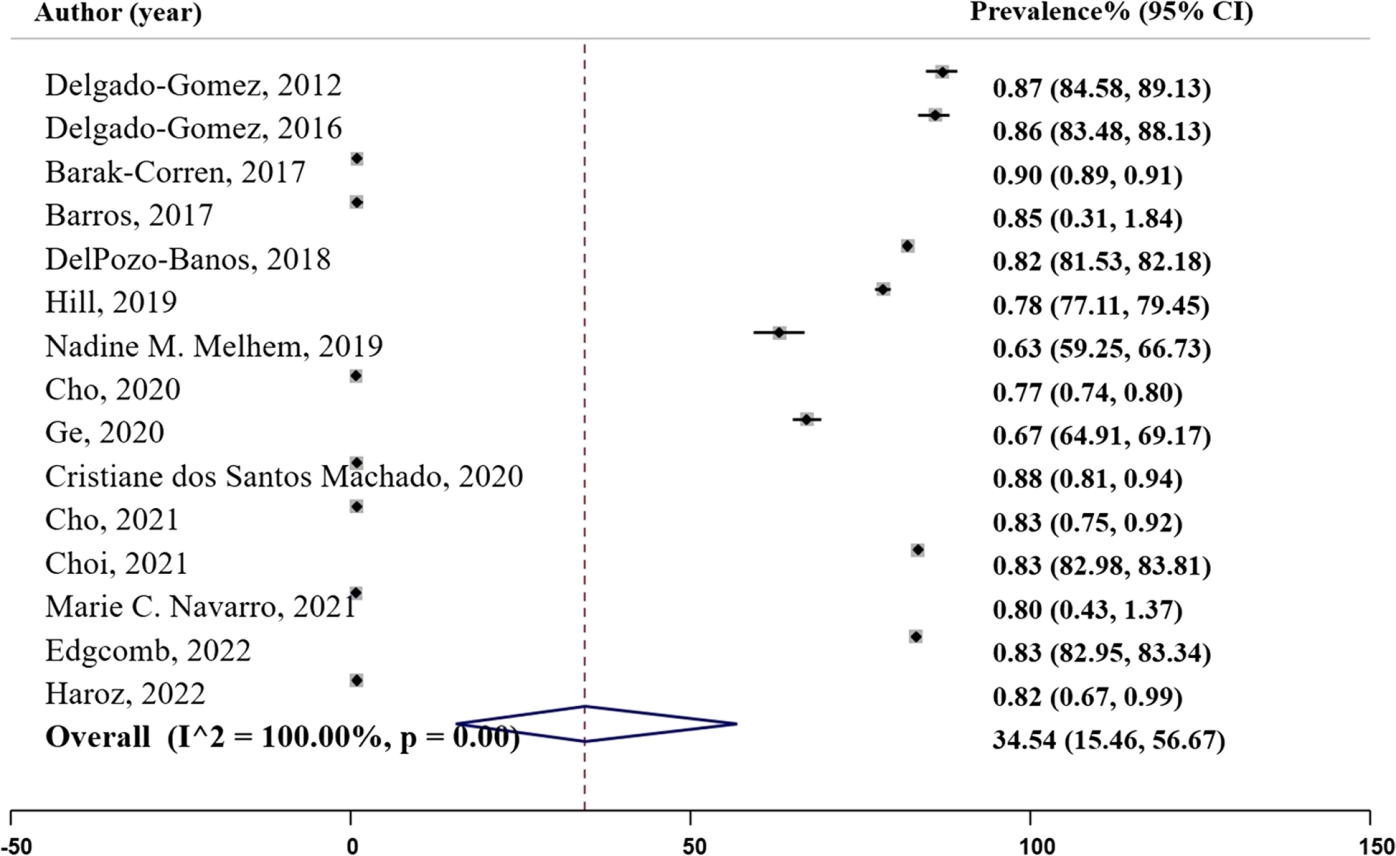
Panel G. Specificity of the machine learning models; N studies = 15

Specificity is a measure that identifies the proportion of actual negative cases correctly identified by the model [28]. In our analysis, fifteen studies reported specificity rates as illustrated in Fig. 8, Panel G. The specificity ranged from 0.63 in Melhem et al.’s (2019) study using logistic regression, to 0.90 in Barak-Corren et al.’s (2017) study using Naive Bayesian classifier. The pooled prevalence of specificity was estimated to be 0.81 (${I}^{2}= 80.31\%;$95% CI: 0.77, 0.86), Table 2.

### Recall

**Fig. 9:**
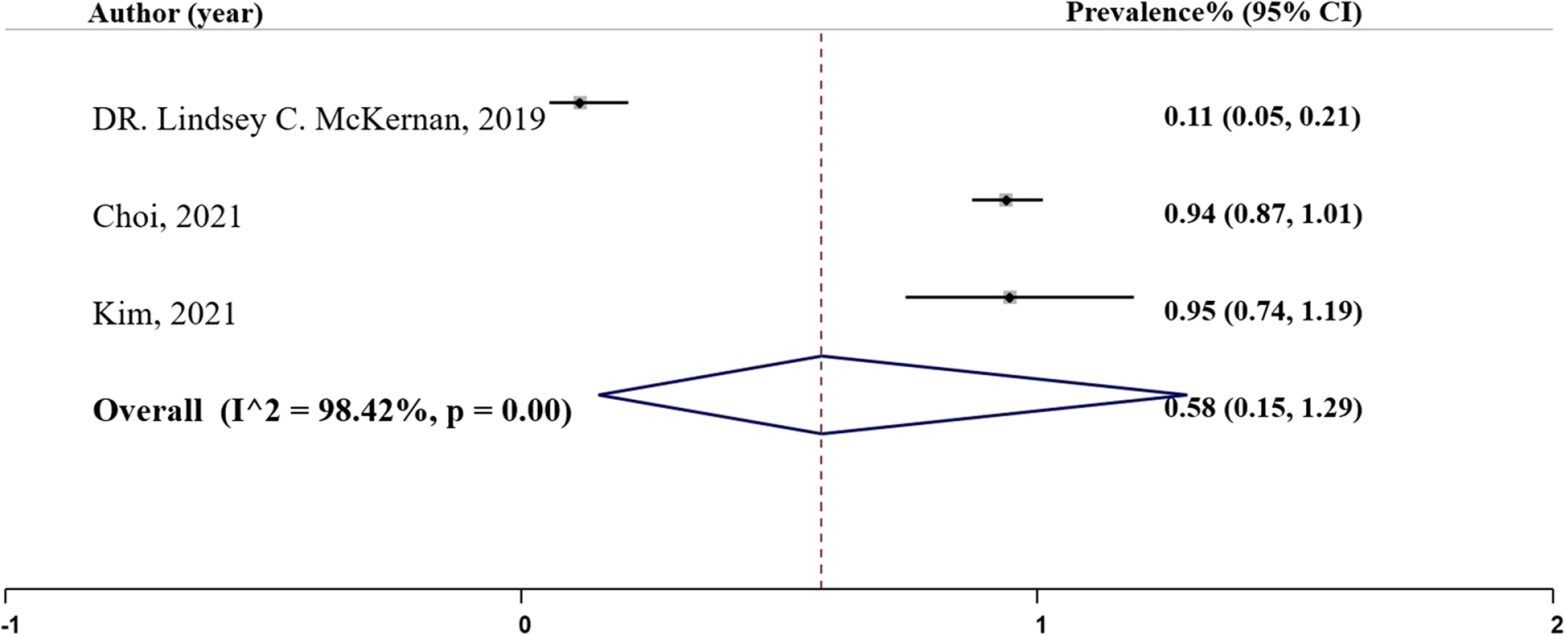
Panel H. Recall of the machine learning models; N studies = 3

Recall is a measure that determines the proportion of true positive cases correctly identified by the model [27]. In our analysis, three studies reported recall rates as depicted in Fig. 9, Panel H, ranging from 0.11 in McKernan et al.’s (2019) study using bootstrapped L-1 penalized regression to 0.95 in Kim et al.’s (2021) study using random forest. The pooled prevalence of recall was estimated to be 0.58 (${I}^{2}= 98.43\%;$95% CI: 0.15, 1.29), Table 2.

### False negative rate

**Fig. 10:**
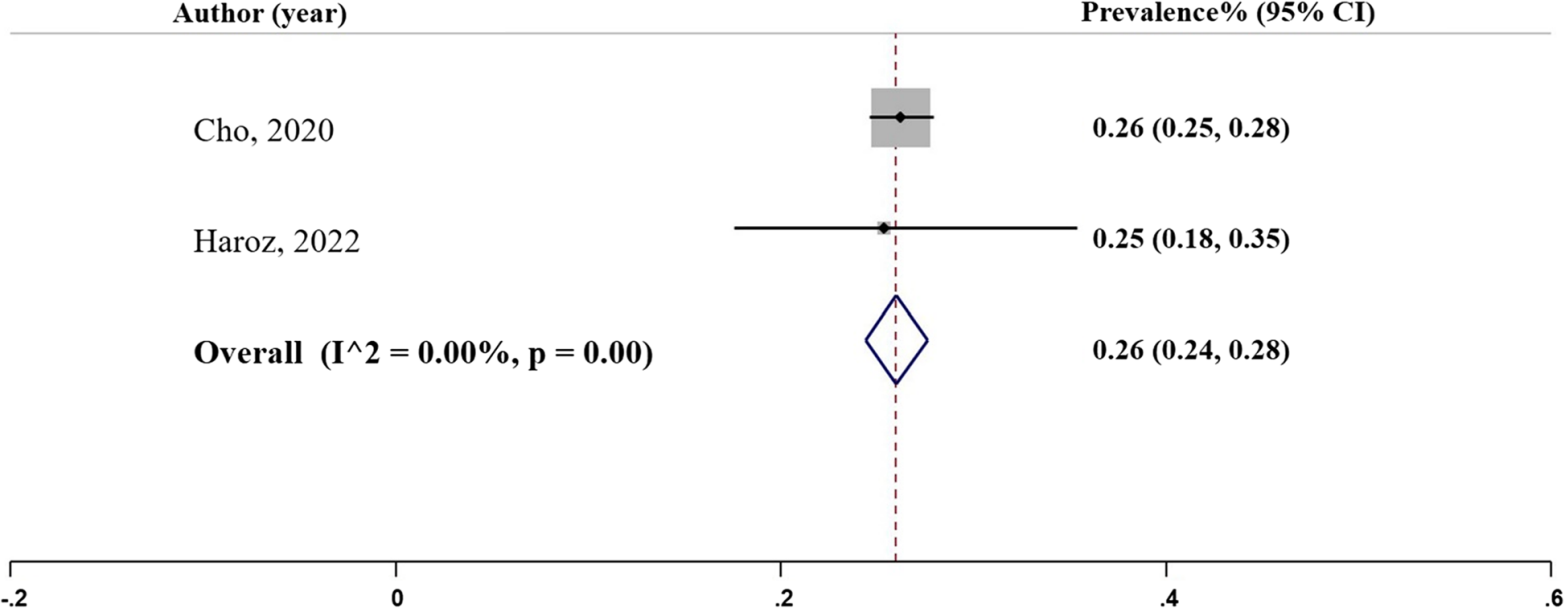
Panel I. False negative rate of the machine learning models; N studies = 2

False negative rate represents the proportion of actual negative cases incorrectly identified by the model [29]. Two studies provided data on false negative rates, with rates that were similar to each other as shown in Fig. 10, Panel I. These studies utilized the random forest and binary logistic regression models. The pooled prevalence of the false negative rate was estimated to be 0.26 (${I}^{2}= 0.001\%;$95% CI: 0.24, 0.28), Table 2.

### Suicide risk factors

In our meta-synthesis analysis, we studied 41 studies in which we identified 261 suicide risk factors. We implemented a rigorous extraction process to identify the most significant risk factors. While some studies presented vast datasets with over 2500 entries of potential risk factors, the focus was on extracting those factors consistently cited as common and important indicators of suicide risk across multiple studies [30, 31]. To ensure robustness, we excluded risk factors reported less than three times, resulting in the compilation of 55 frequently occurring risk factors. We aimed to focus on more prevalent risk factors in the database to enhance the generalizability of the findings to the broader population. Some factors with lower frequencies can introduce noise in the analysis, making it more challenging to identify true patterns. The minimum threshold helped us filter out less relevant factors. This decision was based on a focus group session that included two psychiatrists and one emergency physician. The focus group selected the most common variables that were repeated more than three times based on their scientific knowledge and experience. These factors were categorized into five distinct categories in our study, as outlined in Table 3.

**Table 3:** Frequently occurring suicide categories and risk factors

Category	Suicide risk factor	Frequency
Demographic	Age	**30**
	Sex	**26**
	Marital Status	**12**
	Education	**10**
	Occupation	**10**
	Income	**10**
	Race	9
	Parents’ Psychological State	5
	Parental status	4
	Insurance	4
	Family or Friends’ History of Suicide	4
	Ethnicity	4
	Social Support Status	3
	Immigrant	3
Laboratory & Biomarkers	Urinalysis with Microscopic	3
	POC Pregnancy Urine	3
	Glucose	5
	LDL	3
	Cholesterol	3
	Triglyceride	3
Scales	Hamilton Depression Rating Scale	3
	Patient Health Questionnaire (PHQ)-9	3
Lifestyle	Substance Abuse	**26**
	Alcohol	**12**
	Activation	3
	Physical activity	3
Clinical & Behavioral	Depression	**21**
	Anxiety	**13**
	Mental Disorders	8
	Antidepressant	8
	Bipolar	7
	Other Drug	7
	Personality Disorders	7
	Psychotic Disorders	6
	Impulsivity	6
	Diseases of the Nervous System	5
	Injury	5
	BMI	5
	Mania	4
	Open wound	4
	Mood Disorder	4
	Poisoning	4
	Stress	4
	Schizophrenia	4
	Hopelessness	3
	Self-esteem	3
	Entrapment	3
	Cerebrovascular Disease	3
	AIDS	3
	ADHD	3
	Endocrine	3
	Fear	3

The numbers highlighted in bold indicate suicide factors that occur with higher frequency and have a more pronounced impact

## Discussion

This study employed a systematic review, meta-analysis, and meta-synthesis approach to examine the pooled prevalence of ML outcomes for predicting suicide and provide a comprehensive list of suicide risk factors. The intricate nature of suicide as a behavior is underscored by a diverse array of risk factors, spanning clinical variables to lifestyle influences [32]. Our study adopted a comprehensive approach, employing both qualitative and quantitative methods. Additionally, the study was limited to studies with prospective, retrospective, retrospective cohort, case cohort, case-control, cohort, diagnostic/prognostic, longitudinal, longitudinal cohort, longitudinal prospective, prognostic, prospective cohort, retrospective, retrospective cohort, and randomized control trial designs due to the large number of studies in the final stage, and to ensure methodological rigor. Ultimately, 41 studies were selected for the meta-analysis and meta-synthesis, meeting the quality assessment criteria. Results revealed the neural network (NN) algorithm with the lowest accuracy at 0.70, contrasting with the random forest exhibiting the highest accuracy at 0.94. Furthermore, the XGBoost classifier demonstrated the highest Area Under the Curve (AUC) value, reaching an impressive 0.97. These findings not only contribute to our understanding of suicide risk factors but also highlight the significance of methodological considerations and algorithmic performance in predictive models.

The findings of this study are consistent with previous research conducted by [33, 34] which suggested that ML algorithms and the identification of innovative indicators play a valuable role in predicting suicide and detecting mental health issues. However, these findings contradict the results of [35], which indicated insufficient evidence to support the superior performance of ML over logistic regression in clinical prediction models. The studies included in the analysis that used ML techniques to predict suicidal attempts demonstrated overall good performance on the most commonly used algorithms, namely XGBoost. For example, the AUC values reported in these studies were consistently high, ranging approximately between 0.65 and 0.97. An AUC value of 0.5 indicates a random prediction, while a value of 1 represents a perfect prediction. The AUC values in the range of 0.97 for XGBoost model suggest that the ML models had a high degree of accuracy in classifying individuals with respect to their risk of suicidal attempts. The findings of this study are consistent with previous research conducted by [36] which confirmed acceptable performance of XGBoost algorithm in cognition of patients with major depressive disorder. This result may be due to the fact that XGBoost is an ensemble model that constructs various models to reduce classification errors on each iteration. According to [37], certain ML algorithms, such as support vector machines (SVM) and decision trees (DT), are preferred over others due to their superior performance in predicting suicide risk. Furthermore [38], confirmed that the application of ML techniques to analyze large databases holds great potential for facilitating the prediction of suicide, offering promising avenues for future research. The results of this study align with the findings of [39], which highlighted the ability of ML to enhance suicide prediction models by incorporating a larger number of suicide risk factors. Applicability of these methods in specific patient groups is invaluable. For example [40], indicated that predicting whether a person has a mental illness itself poses a significant challenge. Therefore, if machine learning can offer a new avenue of hope for clinicians, it is commendable. However [41], discovered that although these models have demonstrated accuracy in the overall classification, their ability to predict future events remains limited in the context of suicide prediction models.

Consequently, it is important to note that the performance of ML algorithms can vary depending on various factors, including the quality and size of the dataset, the specific features used as input, the preprocessing steps applied, and the hyperparameters selected for the algorithms. Therefore, the overall performance of these algorithms in predicting suicide showed strong discriminatory power in distinguishing between individuals who are at risk of suicidal attempts and those who are not. Future research should continue exploring and refining ML approaches for suicide prediction, considering these factors to improve the accuracy and reliability of predictions.

The findings of our study revealed that various factors, such as age, sex, substance abuse, depression, anxiety, alcohol consumption, marital status, income, education, low-density lipoprotein (LDL) and occupation, were identified as the most prevalent risk factors based on the analysis of included studies. Age plays a complex role in suicide, with several studies indicating a higher incidence of suicide among middle-aged and older adults. However, it is important to note that age is not the sole factor contributing to suicidal behavior [42, 43]. The prevalence of suicide is exceptionally high among young adults, specifically those aged 15 to 19 as it is a fourth cause of death in the world [44]. Sex is a significant risk factor for suicide. In general, men are more likely to die by suicide than women, but women attempt suicide more often than men. This may be because men are more likely to use lethal methods [42, 45, 46].

According to the meta-synthesis results, there appears to be a significant correlation between substance abuse and depression with suicide. This correlation may be because substance abuse can impair judgment and increase impulsivity. On the other hand, a person who is depressed may experience feelings of hopelessness, helplessness, and despair, which can lead to suicidal thoughts or behaviors. These findings align with the study conducted by [47, 48]. Anxiety as a mental health condition can lead to various negative outcomes, including an increased risk of suicide [49]. Alcohol use can increase impulsivity and decrease inhibitions, leading to risky behaviors such as self-harm or suicide attempts [50, 51]. found that the consumption of alcohol while feeling sad or depressed could indicate suicidal behavior in adolescents who had not previously reported having thoughts of suicide before attempting it.

Marital status is a common suicide risk factor. Researchers have found that married individuals have lower suicide rates than their unmarried counterparts. This trend is observed in both men and women across different age groups and cultures [52]. Low income has been associated with an increased risk of suicide. The reasons for this link are complex and multifactorial, but some possible explanations include limited access to healthcare and mental health services, financial strain, and social isolation [53]. Lower education levels are also associated with higher suicide rates. This may be because lower education-level individuals have fewer job opportunities and may experience more financial stress [53]. In addition to the clinical and demographic factors discussed, it is crucial to recognize the significant role that certain biomarkers and laboratory factors play in the vulnerability to suicide. One notable example is the impact of low serum cholesterol levels, which have been found to significantly heighten the risk of suicide [54]. Some studies have shown that LDL level is an important factor in the incidence of suicide [55]. Moreover, some studies have indicated that individuals who have committed suicide had higher levels of LDL compared to non-attempters [56].

Machine learning (ML) techniques are suitable for predicting suicide risk, overcoming the constraints of traditional methods. However, ML requires sufficient and relevant data to train and validate the early identification of risk factors and suicide prediction. We acknowledge the importance of anticipating and addressing immediate concerns related to suicide in a clinical setting. Due to this, some studies have focused on utilizing certain scales in psychiatric outpatients [57]. However, reliance solely on these scales may instill an unwarranted sense of assurance among healthcare providers. Hence, it is crucial to factor in data availability and the computational demands of handling extensive datasets and intricate models. Our evaluation underscores the proficiency of ML algorithms in uncovering concealed relationships and delivering precise predictions of suicide risk, contingent upon the judicious selection and meticulous evaluation of algorithms. This underscores the indispensable role of ML algorithms in exhaustively analyzing data and pinpointing crucial risk factors, thereby advocating for further exploration in the field. This methodological breadth mirrors the multifaceted nature of suicide risk prediction, enhancing the generalizability of our findings. However, our study may be susceptible to limitations arising from the included studies and the meta-analysis methodology. Additionally, reliance on published literature may introduce publication bias, favoring studies with statistically significant results and potentially skewing overall findings. Furthermore, it is suggested to report τ² and the Q-statistic in future studies to assess heterogeneity. Despite these challenges, our study offers valuable insights into the role of machine learning algorithms in predicting suicide risk and sheds light on important risk factors associated with suicidal behavior. Future research endeavors will continue to tackle these methodological hurdles, striving for enhanced standardization and transparency in study reporting to fortify the reliability and reproducibility of findings in this crucial domain of inquiry.

### Ethical considerations in the use of ML for suicide prediction

Machine learning (ML) for suicide prediction requires the implementation of ethical considerations as the well-being and rights of individuals and the privacy and confidentiality of individuals’ data are crucial. Participants should be fully informed about the study’s purpose, potential risks, and benefits and have the right to withdraw their consent at any time. Understanding and interpreting the factors and variables that contribute to the predictions is important. This transparency is required to gain the trust of both individuals at risk and healthcare professionals. Ensuring that ML algorithms cannot be replaced by human intervention and clinical judgment is important. Human oversight is critical in interpreting and acting upon the predictions made by the algorithms. Healthcare professionals should make informed decisions based on ML predictions, considering the individual’s unique circumstances and context [58].

## Conclusion

Suicide is a complex and multifaceted public health issue with significant implications for individuals and communities. Our study examined the application of ML techniques for predicting suicide risk. Our research findings highlight the diverse performance of ML algorithms in predicting suicide, indicating the need for further investigation and refinement.

Our analysis identified several general risk factors contributing to an individual’s heightened risk of suicide. These factors include age, sex, substance abuse, depression, anxiety, alcohol consumption, marital status, income, education, and occupation. Recognizing that these risk factors interact in complex ways is important, and their presence does not guarantee suicidal behaviour. Nonetheless, understanding and addressing these risk factors can aid in developing targeted prevention and intervention strategies.

While ML algorithms have shown promise in predicting suicide risk, their performance can vary depending on the specific dataset and risk factors being considered. Further studies are warranted to explore using ML algorithms across diverse databases encompassing various risk factors. This would allow for a more comprehensive understanding of the predictive capabilities of ML in different contexts and populations.

Moreover, future research should focus on enhancing the interpretability and explainability of ML models in suicide prediction. Understanding the underlying mechanisms and variables contributing to predictions is essential for effective intervention and decision-making. Additionally, rigorous validation and evaluation of ML algorithms should be conducted to assess their accuracy, generalizability, and potential biases.

To advance the field of suicide prediction using ML, collaboration between researchers, clinicians, and policymakers is crucial. This interdisciplinary approach can foster the development of comprehensive and ethical frameworks for implementing ML algorithms in suicide prevention efforts. Ensuring that ML techniques are used responsibly, prioritizing patient well-being, privacy, and equitable outcomes is imperative.

In conclusion, our study sheds light on the potential of ML algorithms in predicting suicide risk. However, further research is needed to refine and validate these algorithms across different datasets and risk factors. By understanding the complexities of suicide and leveraging the power of ML, we can work towards more effective strategies for suicide prevention and intervention.

## Data Availability

All data generated or analysed during this study are included in this published article.
